# Metabolic and Inflammatory Responses after ERCP

**Published:** 2013-12

**Authors:** Gokhan Adas, Ahu Kemik, Mine Adas, Bora Koc, Emin Gurbuz, Adem Akcakaya, Servet Karahan

**Affiliations:** 1 Bakirköy Dr.Sadi Konuk Training and Research Hospital, Department of Surgery, Istanbul, Turkey;; 2 Istanbul University, Faculty of Medicine, Department of Biochemistry, Istanbul, Turkey;; 3 Okmeydani Training and Research Hospital, Department of Endocrinology, Istanbul, Turkey;; 4 Okmeydani Training and Research Hospital, Department of Surgery, Istanbul, Turkey;; 5 Bezmialem Vakif University, Faculty of Medicine, Department of Surgery, Istanbul, Turkey

**Keywords:** ERCP, inflammatory response, surgical trauma

## Abstract

**Background:**

We aim to evaluate the metabolic and inflammatory responses after ERCP procedure in patients who have common bile duct stones.

**Methods:**

Between September 2009 and October 2010, we studied prospectively 50 patients who diagnosed with common bile duct stones. Our study was included patients who had previously been suspected with common biliary duct stone via radiological and biochemical examinations. We investigated parameters of pro-inflammatory cytokines (IL-1β, IL-6, Il-8, IL-12, IFN-γ, TNF-α), anti inflammatory cytokines (IL-4, IL-10, IL-13), stress hormones (ACTH, cortisol, growth hormone, aldosterone) and acute phase reactant (CRP). All venous blood samples were taken firstly 1hr before endoscopic intervention as a control. After ERCP procedure, venous blood samples were taken two more times, the first in 1hr, the second in 24 hours.

**Results:**

We performed ERCP successfully to 50 patients due to common bile duct stones. All of them had higher serum cytokine levels (*p*<0.01) after an hour and 24 hours later ERCP than before endoscopic intervention except IL-13 level. A significant increase (*p*<0,01) was found in ACTH, cortisol, GH and aldosterone levels 1 hour and 24 hours after ERCP, except GH level (*p*>0.05). CRP level was significiantly increased 1 hour and 24 hours after ERCP.

**Conclusion:**

ERCP procedure is a kind of invasive attempt as known, also causes, with its effects, systemically inflammatory response in the body. This response, mostly not staying at the local stage, becomes systemic inflammatory response. Therefore, before ERCP is performed, the applications of other non-invasive methods of diagnosis are strongly advised.

## INTRODUCTION

Surgical injury induces a systemic endocrine-metabolic response, which includes stimulation of the hypothalamic pituitary-adrenal axis and the sympathetic nervous system. It leads to production of cytokines and acute phase proteins; increases in the levels of stress hormones, loss of muscle protein, greater vascular permeability and changes in white cell count subsets (1-4). This response is proportional to the severity of surgical stress. Local injuries of limited duration are usually followed by functional restoration with minimal intervention. By contrast, major insults to the host are associated with an overwhelming inflammatory response that, without appropriate and timely intervention, can lead to multiple organ failure and adversely impact patient survival (5). Like other conditions associated with tissue injury, such as infection, burns, and trauma, surgery evokes a potent local and systemic inflammatory response manifested by rapid changes in the plasma concentration of various acute-phase proteins and pro-inflammatory cytokines (6).

Endoscopic retrograde cholangiopancreatography (ERCP) is a combined endoscopic and radiographic procedure that allows visualization of the biliary and pancreatic duct systems by direct cannulation of the papilla of vater and retrograde contrast injection using a side viewing duodenoscopy (7, 8). ERCP is also a kind of the minimally invasive surgery. It has a greater potential for procedure related complications than other endoscopic procedures in the upper gastrointestinal tract (7). The magnitude of the metabolic response to ERCP is not known exactly. Metabolic changes are occurred during ERCP and inflammatory cytokines, stress hormones and acute phase reactants may release during this procedure.

This study compared the magnitude of the metabolic response before and after ERCP procedure. Our aim is to find out whether or not a trauma following ERCP, which is known as a minimally invasive surgical procedure, occurs and if occurs to determine the degree of the trauma. We investigated parameters of pro-inflammatory cytokines, anti inflammatory cytokines, some stress hormones and acute phase reactant.

## METHODS

Between September 2009 and October 2010, we studied prospectively 50 patients who diagnosed with common bile duct stones. Our study included patients who had previously been suspected with common biliary duct stone via radiological and biochemical examinations. Initial patient evaluations consisted of history and physical examinations and laboratory findings. All patients were screened by trans-abdominal ultrasound or MRCP scan before the ERCP procedure for suspicion stones in the common bile duct.

### ERCP Procedure

ERCP procedure was applied in the morning after overnight fast. All endoscopies were performed The Day Surgery Center at the Hepatobiliary Department of Surgery at Okmeydani Training and Research Hospital. All patients admitted to surgery service and informed consent for the procedure before ERCP. Intravenous antibiotic (1 gr Ampicilin + Sulbactam) was administered before the procedure and continued until the time of patient discharge. Patients received 2-5 mg of midazolam (Dormicum) intravenously for sedation. Duodenal peristalsis was inhibited by hyocine-n-butyl bromide (Buscopan). All ERCP procedures were done by two experienced endoscopists with Fujinon duodenescope (250X-T5). Precut papillotomy was done routinely in ERCP procedure. Additionally; we separated patients to three groups according to the number of cannulation (easy 1-5, medium 5-10, difficult≥10) (9, 10). Biliary stones were removed with dormia basket or balloon, if they were observed during ERCP. Information about concomitant medications, clinical history, duration of the endoscopic procedure, total time, complications, and main pathology were recorded during the ERCP procedure. All patients admitted to surgery service after procedure and observed. In addition to study parameters blood cell and amylase levels were routinely evaluated at the observation period. In the case of absence of increases in clinical and biochemical parameters patients discharged 2-4 hours after the procedure.

### Investigated Parameters

All venous blood samples were taken initially 1hr before endoscopic intervention. After ERCP was done, venous blood samples were taken two more times, the first in 1 hr, the second in 24 hours after the ERCP procedure.Blood samples were allowed to coagulate at room temperature for 30 min and centrifuged at 10.000 g for 15 minutes. Serum was separated and stored -70°C until assay. We investigated parameters of pro-inflammatory cytokines (IL-1β, IL-6, IL-8, IL-12, IFN-γ, TNF-α), anti inflammatory cytokines (IL-4, IL-10, IL-13), stress hormones (ACTH, cortisol, growth hormone (GH), aldosterone) and acute phase reactant (CRP). All cytokines parameters were determined by enzyme linked immune-absorbent assay (ELISA). ACTH, cortisol, aldosterone, GH and CRP were determined by spectrophotometric analyzed.

Our exclusion criteria were such as acute cholangitis, malign obstructive jaundice (cholongiocancer, periampuller tm, pancreas tm) and emergency ERCP namely acute biliary necrotized pancreatitis.

### Statistical Analysis

Multivariate test (Pillai’s) was used to evaluate statistical analysis. Comparison was evaluated by Bonferroni test. Differences were determined using the least significant differences and *p*<0.05 was considered to be significant. Results were expressed as mean ± SD.

## RESULTS

During the study period, 50 patients (29 male, 21 female) with a mean age of 58 years (range, 26 to 86) underwent ERCP procedure for common bile duct stones. The ERCP procedure was found to be technically successful in 100% of patients. No mortality and major complications were observed in our study. In the patients, minor complications were seen in two patients (one mild stage pancreatitis and one mild stage bleeding) (11, 12). We recognized the patients acute pancreatitis if the amylase levels increased three times to baseline levels (13) and accompanied with clinical pancreatitis signs. In our study, the patient diagnosed as acute pancreatitis while the amylase level increased 6 times (750 U/L) to normal range (range 25-125U/L). Two patients could be treated medically. The patient with bleeding and diagnosed as acute pancreatitis discharged second day and fifth day after the admission, respectively. Succeed of cannulation of bile duct during ERCP procedure; 28 (56%) of the patients were mild, 18 (36%) of patients were middle and remaining of the group 8% was difficult cannulation. Microlitiazis were obtained in 23 (46%) patients, macrolitiazis in 25 (50%) patients, there were no any clue of stone in 2 (4%) of the patients. Characteristics of patients and complications were given in Table 1.

**Table 1:** Characteristics of patients and underwent ERCP procedure for common bile duct stones


Patients (n:50)	
Age (years)	58 (26-86)
Gender M/F	29/21
Complications	2
Acute pancreatitis	1
Mild bleeding	1
Need for surgery	-
Deaths	-
Time of ERCP procedure (means) (minutes)	23 (min 15 - max 47)
Number of Cannulation	
Easy (1-5)	28 (56%)
Mild (5-10)	18 (36%)
Difficult (10>)	4 (8%)
Microlithiasis patients	23 (46%)
Macrolithiasis patients	25 (50%)
No stone patients	2 (4%)

Number of cannulations were given according to cannulation attempts. Values are mean, minimum and maximum

### Metabolic Response of Hormone Levels

Significant differences (*p*<0.01) were found in hormones of ACTH, cortisol, GH, aldosterone that was taken one hour after ERCP procedure. Also we did not find any differences in hormones of GH 24 hour after the procedure. Significant differences (*p*<0.01) were found in hormones including ACTH, cortisol, aldosterone 24 hours after ERCP. The results are presented in Table 2.

**Table 2:** Plasma concentrations of hormone levels, acute phase reactant (CRP) level before and after ERCP procedure

Stress hormones and acute phase reactan levels	1h before ERCP (n=50)	1h after ERCP (n=50)	24h after ERCP (n=50)	P 1h after ERCP	P 24h after ERCP

**ACTH (pgr/ml)**	73 (61-82)	91 (78-101)	79 (63-96)	*p*<0.01	*p*<0.01
**Cortisol (nmol/l)**	244 (189-291)	321(226-389)	312(241-388)	*p*<0.01	*p*<0.01
**GH (mU/lt)**	122 (9-178)	174(141-197)	128(98-186)	*p*<0.01	NS
**Aldosterone (ngr/dl)**	9 (7-11)	13 (11-16)	11 (7-16)	*p*<0.01	*p*<0.01
**CRP(mlgr/dl)**	1,8 (1-3)	4 ( 2-5)	5 (1-15)	*p*<0.01	*p*<0.01

Values are mean, minimum and maximum. *P*<0.05 was regarded as a significant. NS, non significant.

### Acute Phase Reactant

There were significant differences between CRP levels compare to before and one hour and 24 hours after ERCP.

### Metabolic Response of Cytokines

We found significant differences (*p*<0.01) in pro-inflammatory cytokines IL-1γ, IL-6, IL-8, IL-12, IFN-γ, TNF-α and found anti inflammatory cytokines IL-4, IL-10, IL-13 at one hour after ERCP procedure compare to before endoscopic intervention. There were significant differences between pro-inflammatory and anti inflammatory cytokines after the procedure (1 h-24 h) compare to prior ERCP procedure except IL-13 level. The mean values of each cytokine level (min-max) were given in Table 3.

**Table 3:** Plasma concentrations of pro-inflammatory and anti inflammatory cytokine levels before and after ERCP procedure

Cytokine levels	1h before ERCP (n=50)	1h after ERCP (n=50)	24h after ERCP (n=50)	P 1h after ERCP	P 24h after ERCP

**Pro-inflammatory cytokine levels**					
**TNF-α (pg/ml)**	15 (13-16)	17 (15-18)	17 (14-18)	*p*<0.01	*p*<0.01
**IL1-β (pg/ml)**	11 (9-12	13 (11-16)	13 (11-15)	*p*<0.01	*p*<0.01
**IL-6 (pg/ml)**	86 (76-92)	93 (86-100)	92 (88-96)	*p*<0.01	*p*<0.01
**IL-8 (pg/ml)**	20 (14-22)	25 (20-28)	24 (20-28)	*p*<0.01	*p*<0.01
**IL-12 (pg/ml**	27 (19-30)	31 (26-34)	25 (19-31)	*p*<0.01	*p*<0.01
**Anti-inflammatoryvcytokine levels**					
**IL-4 (pg/ml)**	17 (14-24)	23 (20-27)	24 (20-28)	*p*<0.01	*p*<0.01
**IL-10 (pg/ml)**	9 (7-11)	13 (10-19)	14 (10-20)	*p*<0.01	*p*<0.01
**IL-13 (pg/ml)**	13 (10-16)	16 (14-20)	12 (10-16)	*p*<0.01	NS

Values are mean, minimum and maximum. *P*<0.05 was regarded as a significant. NS, non-significant.

## DISCUSSION

Surgical trauma induces an inflammatory state characterized by the release of pro-inflammatory cytokines and acute phase proteins. The inflammatory response to injury involves an interplay among hormones (catecholamines, adrenocorticotropic hormone, cortisol, glucagon), cytokines and other cellular products such as proteases, free radicals, eicosanoids, acute phase reactants and growth factors (6, 14). ERCP procedure is a kind of minimally invasive method and has been used more common for treatment of choledocholithiasis. In literature, studies on the trauma for minimally invasive surgery are mostly related with laparoscopic biliary surgery. Currently no data is available regarding surgical stress response and immune competence following ERCP procedure. In this study we wanted to show the metabolic response to trauma after ERCP interventions. We determined that the metabolic response to trauma was started as local response but enlarged to metabolic condition. Although there have been a lot of study for metabolic inflammatory changes in minor and major operations and also minimally invasive attempts in literature, there has not been any wide spectrum study for ERCP procedure.

The acute phase responce, is initiated locally at the site of the surgical trauma by macrophages and monocytes, which release cytokines (15). They are released transiently to modulate immune or repair process by controlling cellular growth, differentiation, metabolism and protein synthesis (16). Although some cytokines play pro-inflammatory role in the body, some typesof these cytokines were evaluated as antiinflammatory. The pro-inflammatory mediators are IL-1β, IL-6, IL-8, IL12 and TNFα, whereas the anti-inflammatory cytokines are IL-4, IL-10, IL-13 (14-20). TNF-α, IL-1, IL-6 and IL-8 are major mediators of the acute phase response in humans (21, 22). There is little knowledge about local response and importance in the morbidity after trauma. It has been hypothesized that effects of contusion release cytokines that are constitutively present from damaged cells and inflammatory cells in the vicinity of the trauma (23). Plasma levels of acute phase proteins such as C-reactive protein, the most widely measured marker of the acute phase response, and the pro-inflammatory cytokines IL1, IL6, IL8 and TNF-α are transiently increased following significant tissue injury. The chief stimulator of the production of most acute phase proteins is IL-6 and high plasma concentrations could be measured after trauma and surgical interventions (16, 24, 25). IL-6 levels are early and sensitive markers of tissue damage because they rise in proportion to the surgical trauma and associated injury. Additionally, elevations in IL-6 levels have been correlated with the subsequent clinical development of major complications (26). The peak concentrations of IL-6 after operations are reported to occur between 3 h and 24 h (27). The generation of acute phase protein is well-recognized response to tissue injury. The CRP is a key marker acute-phase protein that has a consistent response and provides a dependable screening test overall for acute phase reactants (26, 28). The CRP rise approximately 4 to 12 hours after surgery and peak at 24 to 72 hours (26). In this study, significant increases in pro and antiinflammatory cytokine levels were observed after ERCP procedure. Especially, when pre-ERCP results were compared with post-ERCP’s, there were significant differences between each other (*p*<0.01). Moreover, the post-ERCP results of CRP level after 24 h also showed significant differences when compared with pre-ERCP’s. The study showed that not only local inflammatory also systemic response to ERCP procedure was obtained as a minor surgery.

The stress response is also the name given to the hormonal and metabolic changes, which follow injury or trauma (19, 29). The effect of endocrine response to surgery is an increased secretion of catabolic hormones. The stress response developed as a survival mechanism, which allowed injured animals to sustain themselves until their injuries were healed (30). As to the results of responses of catabolic hormones to ERCP procedure, we demonstrated that there were emerged significantly increased hormone levels when compared with pre and post ERCP procedures in that all the hormone levels in the ERCP after the 1st hour were significantly increased (*p*<0.01). In addition, the levels of cortisol, aldosterone and ACTH after ERCP in the 24th hour were significantly high (*p*<0.01). Our results, the initial local effect and subsequent systemic over flow of inflammatory cytokines from the damaged area (periampuller area) may further induce systemic liberation of cytokines after ERCP procedure. The sphincterotomy is increased the systemic response to trauma according to our consideration. Also cannulation, especially recurrent attempts and difficult interventions were improved the metabolic response related with trauma. The number of cannulation attempts and the statistical analyze of the time of ERCP and metabolic response process are the limitations of our study. Generally; there is a correlation between the complications and increases of the number of cannulation attempts and prolongation of invasive procedure (9, 10). The number of case was limited our study for this comment. This conditions were limited our study.

In literature, studies on ERCP after trauma are mostly associated with ERCP after pancreatitis. Some parameters might reflect ERCP-induced damage, such as the number of cannulation attempts of the papilla of vater or the duration of the procedure. Oezcueruemez-Porsch *et al*. showed that three clinical parameters (time of endoscopy, number of cannulation attempts, and pain) were significantly correlated with peak values of IL10, IL6 correlated with pain and the duration of the endoscopy (31). Messmann *et al*. found elevated concentrations of C-reactive protein and the pro-inflammatory cytokine IL6 in the nine patients with ERCP-induced pancreatitis within hours of the procedure (32). The obvious question is as follows: Do all ERCPs or only ERCP after pancreatitis result in trauma? According to study results by Oezcueruemez-Porsch *et al*. and Messmann *et al*. some parameters are high in patients with post-ERCP pancreatitis. Our results may bring up a new discussion since ERCP leads to a trauma at a certain level in the human body in all cases in addition to their results. Therefore, more research and a wider case study are required on this field.

In conclusion, ERCP is elevated the systemic metabolic response to trauma more than we expected, in our consideration. This response, mostly not staying at the local era, becomes systemic conditions in the body. This metabolic response can be reached the peak levels when the important complications were occurred. We think that non invazive radiologic techniques (such as MRCP) is done for most of diagnostic purpose and ERCP is only used for therapeutic purpose.
